# STRPsearch: fast detection of structured tandem repeat proteins

**DOI:** 10.1093/bioinformatics/btae690

**Published:** 2024-11-18

**Authors:** Soroush Mozaffari, Paula Nazarena Arrías, Damiano Clementel, Damiano Piovesan, Carlo Ferrari, Silvio C E Tosatto, Alexander Miguel Monzon

**Affiliations:** Department of Biomedical Sciences, University of Padova, Padova 35121, Italy; Department of Biomedical Sciences, University of Padova, Padova 35121, Italy; Department of Protein Science, KTH Royal Institute of Technology, Stockholm SE-10691, Sweden; Department of Biomedical Sciences, University of Padova, Padova 35121, Italy; Department of Biomedical Sciences, University of Padova, Padova 35121, Italy; Department of Information Engineering, University of Padua, Padova 35121, Italy; Department of Biomedical Sciences, University of Padova, Padova 35121, Italy; Institute of Biomembranes, Bioenergetics and Molecular Biotechnologies, National Research Council (CNR-IBIOM), Bari 70126, Italy; Department of Information Engineering, University of Padua, Padova 35121, Italy

## Abstract

**Motivation:**

Structured Tandem Repeats Proteins (STRPs) constitute a subclass of tandem repeats characterized by repetitive structural motifs. These proteins exhibit distinct secondary structures that form repetitive tertiary arrangements, often resulting in large molecular assemblies. Despite highly variable sequences, STRPs can perform important and diverse biological functions, maintaining a consistent structure with a variable number of repeat units. With the advent of protein structure prediction methods, millions of 3D models of proteins are now publicly available. However, automatic detection of STRPs remains challenging with current state-of-the-art tools due to their lack of accuracy and long execution times, hindering their application on large datasets. In most cases, manual curation remains the most accurate method for detecting and classifying STRPs, making it impracticable to annotate millions of structures.

**Results:**

We introduce STRPsearch, a novel tool for the rapid identification, classification, and mapping of STRPs. Leveraging manually curated entries from RepeatsDB as the known conformational space of STRPs, STRPsearch uses the latest advances in structural alignment for a fast and accurate detection of repeated structural motifs in proteins, followed by an innovative approach to map units and insertions through the generation of TM-score profiles. STRPsearch is highly scalable, efficiently processing large datasets, and can be applied to both experimental structures and predicted models. In addition, it demonstrates superior performance compared to existing tools, offering researchers a reliable and comprehensive solution for STRP analysis across diverse proteomes.

**Availability and implementation:**

STRPsearch is coded in Python. All scripts and associated documentation are available from: https://github.com/BioComputingUP/STRPsearch.

## 1 Introduction

Tandem Repeat Proteins (TRPs) represent a diverse group of proteins featuring repetitive sequence motifs (Kajava and Tosatto 2018). A specialized TRP subset, known as Structured Tandem Repeat Proteins (STRPs) (Monzon *et al.* 2023) is distinguished by the conservation of specific structural motifs rather than mere sequence repetition. In STRPs, the repetitive units are the fundamental structural elements that collectively constitute repeat regions (Di Domenico *et al.* 2014). The proposed classification by Kajava (2012) categorizes tandem repeats into five different classes, based on their architectural arrangement and the length of their constituent units. Recent predictions suggest that 50.9% of proteins across all kingdoms of life are composed of at least one TRP region, with a particular enrichment of TRPs in Eukaryotes (Delucchi *et al.* 2020).

TRPs have been shown to be involved in many biological functions and activities. For example, DNA sliding clamps are TRPs which play an essential role in DNA replication (Arrías *et al.* 2023) while leucine-rich repeats (LRRs) make up the extracellular domains of toll-like receptors (TLRs) involved in host immune responses (Leulier and Lemaitre 2008). In recent years, their significance has garnered increasing attention, owing to their implications in health (de Wit *et al.* 2011, Fournier *et al.* 2013) and their application in protein design (Höcker 2014, Brunette *et al.* 2015, Wu *et al.* 2023). On the other hand, with the steady growth of the Protein Data Bank (PDB), storing >217 000 (March 2024) experimental protein structures, and the huge amount of protein structural models from the recent structure prediction methods such as AlphaFold (Jumper *et al.* 2021) and RoseTTAFold (Baek *et al.* 2021), the scientific community has an unprecedented volume of protein structure data available. This challenges state-of-the-art methods dealing with protein structures.

RepeatsDB (Clementel *et al.* 2024) is the main repository of STRPs annotation and classification. Through manual curation of STRPs on experimental structures, each entry is classified by precisely identifying regions, units, and insertions, including the determination of their position and range within the protein structure. As an outcome of this curation effort, RepeatsDB can serve as a ground-truth for the development and fine-tuning of computational tools designed for the study and analysis of STRPs.

Different predictors have been developed for the automatic TRP detection from sequence or structure (Delucchi *et al.* 2021, Kamel *et al.* 2021). Particularly, tools such as TAPO (Do Viet, Roche and Kajava 2015) and RepeatsDB-lite (Hirsh *et al.* 2018) aimed to detect repeated regions and units from protein structures. The main limitations of these tools include issues with code availability, long running times, poor documentation, lack of ongoing maintenance, and the inability to handle large datasets or large protein structures. This represents a bottleneck for large-scale STRP detection, given the vast amount of structural data available, e.g. in the PDB and AlphaFoldDB (Varadi *et al.* 2022). Here, we present STRPsearch, a fast method to accurately detect STRPs from protein structures. STRPsearch combines the latest advances in protein structure similarity detection with manually curated STRP data from RepeatsDB (Clementel *et al.* 2024). Its design and implementation allow the user to efficiently process large datasets, a simple output format, and easy integration into any bioinformatics pipeline. In addition, STRPsearch is easily extensible and allows the incorporation of new curated data to enhance the detection of novel STRPs.

## 2 Methods

The algorithm requires one primary input which is a protein structure as the query. It then utilizes two structural repeat libraries to identify repeated structural motifs within the input structure. The libraries are built upon the reviewed entries in the RepeatsDB (dated 2023-05-03), comprising an extensive dataset of PDB chains in which repeat regions and units have been manually curated. This represents a sample of the conformational space and diversity of STRPs. The algorithm uses this data through two main libraries: the Tri-Unit-Library (TUL) and the Representative-Unit-Library (RUL). Each library consists of 2460 proteins (with different UniProt IDs), 9121 PDB chains, and 9502 repeat regions, all manually classified by class and topology according to Kajava’s classification (Kajava 2012).

The “representative unit” is a single repeat unit in a repeat region that exhibits the maximum structural similarity, measured by TM-Score (Zhang and Skolnick 2005), to other units within the same region, and is stored in the RUL. Based on the representative unit's position within the region, the two adjacent units (i.e. N- and C-terminal to the RUL) together form a tri-unit structure that is then trimmed and stored in the TUL.

**Figure 1.**
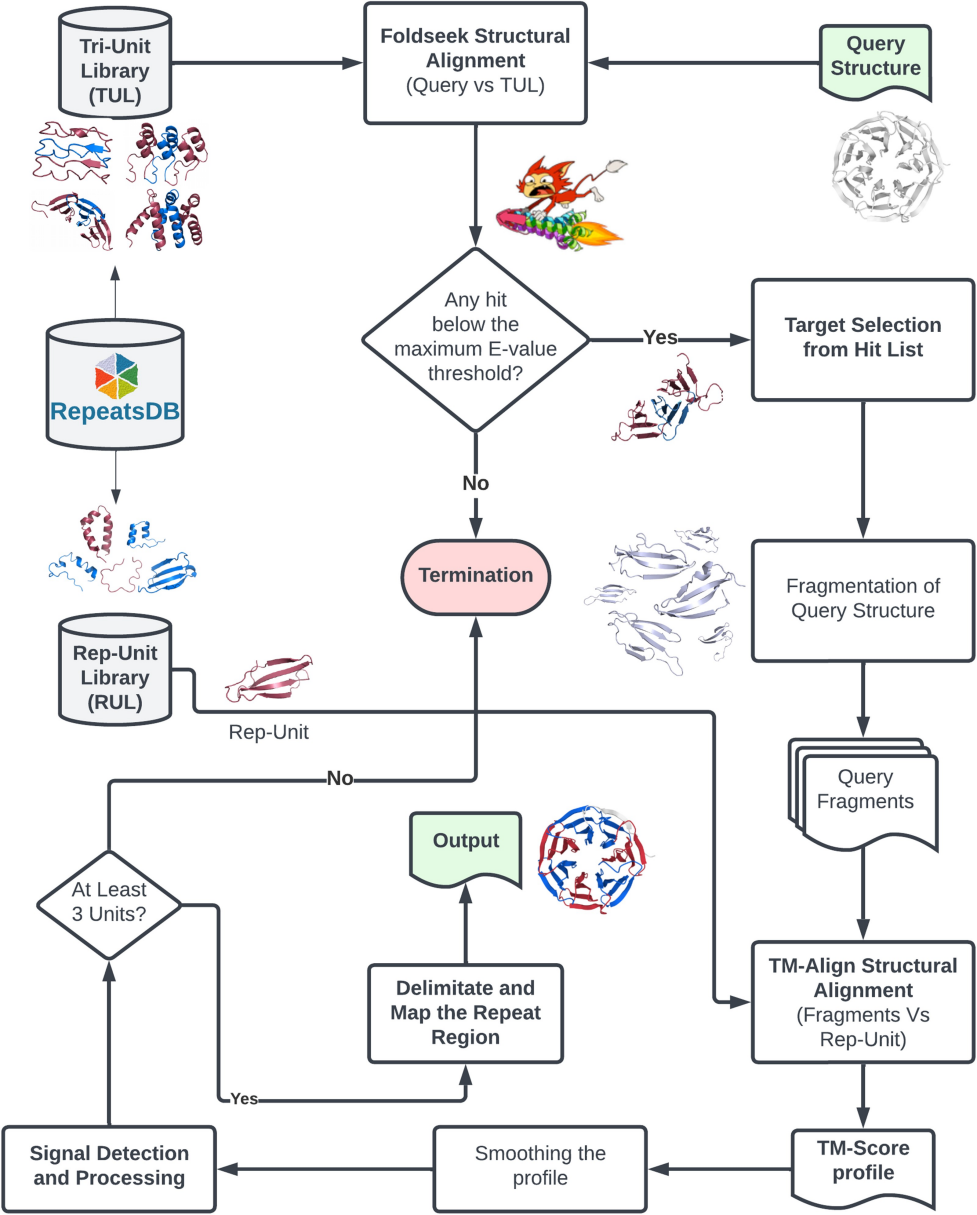
Flowchart of the STRPsearch software method. The query structure is first aligned with the TUL using Foldseek; hits with the lowest E-values are selected for the next step. The query structure is then fragmented into pieces that match the size of the selected hits and aligned with the associated Rep-Unit, retrieved from the RUL, using TM-align for pairwise structural alignment. TM-scores are plotted against the starting residues of the query fragments to illustrate relative structural similarity across the entire query structure. SciPy's signal processing toolbox is used to smooth the TM-score profile and detect peaks. These peaks, which indicate potential repeat unit positions, are interpreted by the software to map the repeat regions. The final output is visualized in PyMOL, with repeat units colored alternatingly.

In the first step, the algorithm searches for repeated structural motifs in the query structure. This involves structurally aligning the query structure against each tri-unit structure in the TUL. Foldseek (van Kempen *et al.* 2024) is used to align the query structure against a customized structure database containing TUL structures. By the end of this stage, the most probable hit based on the E-value of query-target structural alignment pairs is selected. If multiple hits with different repeat types (class and topology) are identified for the same query structure, two or more hits will be chosen according to the classification of the most probable targets (i.e. lowest E-value). For each hit, the representative repeat unit associated with the target is retrieved from the RUL and aligned across the entire length of the query structure using a sliding window approach. This method, which increments by a single residue at each step, aims to improve resolution and accuracy (Fig. 1). To achieve this goal, the query structure is fragmented into pieces matching the length of the target representative unit (Rep-unit). These fragments are then pairwise aligned to the representative unit using TM-align (Zhang and Skolnick 2005), which measures structural similarity through TM-scores. The TM-scores are recorded and plotted against the starting residue numbers of the query fragments to generate a TM-score profile for each residue. These profiles illustrate the variations in structural similarity between the query and representative unit along the full length of the query structure (Fig. 1).

When repeat regions are present, the alignment of the representative unit with similar repeat units in the region produces periodic peaks in the TM-score profile. To accurately identify these peaks and map the integral components of the repeat region based on their positioning, the TM-score profile, which could be interpreted as a graph, undergoes smoothing adjusted to the length of the representative unit. This optimization enhances the performance of peak detection algorithms. Subsequently, using SciPy’s signal processing toolbox, peaks are identified and the integral components are mapped. If at least three adjacent repeat units are identified, a repeat region is mapped onto the query structure, and the class and topology of the representative repeat unit is assigned to this region (Fig. 1).

Two key parameters that can be customized via the command line interface are “max_eval” and “min_height.” The “max_eval” parameter denotes the upper threshold of E-values for hits identified by Foldseek in the initial phase of the algorithm. A higher “max_eval” leads to an increased incidence of false positive results and conversely, lower values can increase the occurrence of false negatives. The “max_eval” parameter is set to 0.1 by default, maximizing the F1-score and achieving the best balance between precision and recall (Supplementary Fig. S2). The “min_height” parameter represents the minimum allowed peak height detected by the peak detection method in the TM-score profile, indicating the potential positions of repeat units. The default value for “min_height” is optimized based on the average structural similarity observed among units of different repeat types in RepeatsDB (Supplementary Fig. S3). Other parameters used by default for the peak detection method are optimized using a grid search.

To evaluate the STRPsearch performance, RepeatsDB served as a reference with 2002 unique protein sequences harboring STRPs from six major types (i.e. Alpha-solenoids, Beta-solenoids, Alpha/Beta-solenoids, Beta-propellers, TIM-barrels, and Beta-barrels). A manually curated negative dataset consisting of 1737 non-STRP structures from unique proteins was used to assess false positive and true negative predictions. Subsequently, both the positive and negative datasets were subjected to clustering at 30% sequence identity using BLASTClust (Altschul *et al.* 1990), and one representative entry from each cluster was randomly selected. This process led to a reduction in the size of both the positive and negative datasets, resulting in 1225 positive and 1218 negative structures (see Supplementary Dataset S1 and S2). The evaluation strategy used a 5-fold stratified cross-validation, allowing the algorithm access to 80% of the positive structures as the template conformational space, while the remaining 20%, in conjunction with a non-overlapping 20% segment of the negative dataset used for validation.

## 3 Results and discussion

STRPsearch is developed in Python version 3.8 exploiting various libraries such as Biopython and SciPy. The tool utilizes Foldseek for aligning the query structure with the TUL and TM-align for aligning query fragments with the corresponding Rep-unit. PyMOL is used to generate session files with colored units and insertions. These external tools are containerized using Docker and integrated within a Conda environment for smooth operation. The source code for STRPsearch is distributed under the GPL license. To operate, STRPsearch requires only an input structure or a PDB/UniProt ID and runs with default parameters.

### 3.1 Application on protein structures

There are three alternatives to execute STRPsearch. The first involves querying a protein structure by providing the input file formatted as PDB/mmCIF, with the option to query either a specific chain or all chains in the structure. Alternatively, users can specify the PDB accession number, allowing the software to automatically download and query a specific chain or all chains in the PDB structure. As a third option, STRPsearch can directly download and query an AlphaFold model by indicating the UniProt accession number.

Upon identification of STRPs, the STRPsearch output includes four components for each identified repeat region: (i) a JSON formatted file containing the classification of the associated repeat region and the boundaries of units and insertions (if they exist), (ii) the trimmed structure of the repeat region in PDB format, (iii) a PyMOL (Schrödinger, LLC 2015) session of the repeat region colored based on units and insertions, and (iv) a TM-score profile per residue, highlighted with the position and range of the repeat units.

### 3.2 Performance evaluation

The cross-validation results indicate that STRPsearch performs consistently well. On average, the tool correctly detects about 80% of all STRP structures, with a standard deviation of 1.9%. For the negative dataset, approximately 10% of non-STRP structures were incorrectly predicted as STRPs, with a standard deviation of 2.57% (refer to Supplementary Tables S3 and S4). When assessing the tool's ability to identify residues within STRP regions, STRPsearch achieved an average accuracy of 88% [(TP + TN)/(TP + FP + TN + FN)], with a precision of 91% [TP/(TP + FP)], a recall (sensitivity) of 91% [TP/(TP + FN)], and an F1-score of 90% [2 * TP/(2 * TP + FP + FN)]. All these metrics showed minimal variance, with standard deviations close to 0.01 (see Supplementary Table S5). Overall, the algorithm demonstrates strong performance in accurately identifying nearly all repeat regions.

### 3.3 Benchmarking

STRPsearch was evaluated against RepeatsDB-lite and TAPO, two web-based tools for STRP identification. This comparative assessment focused on evaluating the ability to differentiate between STRPs and non-STRPs. The evaluation was conducted using a dataset of 244 positive structures and 244 negative structures. The positive structures were randomly selected, ensuring a balanced representation of the main repeat types. As shown in Table 1, while RepeatsDB-lite and TAPO demonstrate relatively high recall rates, STRPsearch outperforms in other performance metrics. A key advantage of STRPsearch is its high specificity (true negative rate) of 0.87 compared to 0.49 for RepeatsDB-lite and 0.58 for TAPO. This indicates fewer false positives, as detailed in Supplementary Figs S5 and S6. When analyzing performance across the six major repeat types, STRPsearch shows excellent recall in detecting closed repeats such as Beta-propellers, TIM-barrels, and Beta-barrels, with few false negatives, as shown in Supplementary Table S6 and Supplementary Figs S7 and S8. All tools perform comparably well in identifying alpha-solenoids and alpha/beta-solenoids STRPs, with similar recall values and few false positives (Supplementary Table S6, Supplementary Figs S7 and S8). However, in detecting beta-solenoids, TAPO has a clear advantage with a recall of 0.92, while STRPsearch shows the lowest recall in this category, suggesting an area for future improvement. This may be due to insufficient representation of beta solenoids in the TUL and FoldSeek accuracy, given the high sequence and structural variability of this fold.

**Table 1.** Comparative STRP identification evaluation.^a^

Method	Accuracy	Precision	Recall	F1-score
STRPsearch	0.85	0.87	0.83	0.85
RepeatsDB-lite	0.66	0.62	0.82	0.71
TAPO	0.74	0.68	0.91	0.78

a Performance evaluation is presented for each method using a dataset comprising 488 structures, with an equal number of positive and negative instances. Underlined values represent the highest among the three methods. See Section 3.2 for details on the measures used.

Comparison of execution times indicates notable differences. STRPsearch processed each entry in an average time of 9 s, with a standard deviation of 9 s. TAPO had an average of 26 s per entry, with a standard deviation of 55 s while RepeatsDB required 190 s on average, with a standard deviation of 216 s (Supplementary Fig. S9).

### 3.4 Running on PDB and AlphaFoldDB model organism proteomes

Running the software on the PDB, 216 478 protein structures (dated 20/02/2024) resulted in the detection of 15 947 putative STRPs, corresponding to 4147 unique protein sequences. In another analysis, on AlphaFoldDB structural models for 48 organisms, totaling 564 446 proteins, STRPsearch identified 40 149 putative STRPs. While computational runtime is highly correlated with protein structure length, for proteins of a length around 500 residues, the average execution time was 35 s with a standard deviation of 20 s.

## 4 Conclusions/summary

We presented STRPsearch, a software designed for fast and accurate identification, classification, and mapping of structural tandem repeats in protein structures. By exploiting the manually curated entries in RepeatsDB as ground-truth and using the latest computational advances in the field, STRPsearch outperforms similar tools with improved reliability, accuracy, and speed. This makes STRPsearch a valuable stand-alone tool for the identification and further analysis of STRPs that could easily be applied to large protein structure databases.

## Supplementary Material

btae690_Supplementary_Data
