# Supplementary material for: STRPsearch: fast detection of structured tandem repeat proteins
Source: Bioinformatics. 2024 Nov 18;40(12):btae690. doi: 10.1093/bioinformatics/btae690 (PMC11645253; doi:10.1093/bioinformatics/btae690)
Supplement: btae690_Supplementary_Data [file btae690_supplementary_data.zip › 3.pdf]

STRPsearch: fast detection of structured tandem repeat proteins

Soroush Mozaffari[1], Paula Nazarena Arrías[1,2], Damiano Clementel[1], Damiano Piovesan[1], Carlo Ferrari[3], Silvio C. E. Tosatto[1,4*], Alexander Miguel Monzon[3,*]

[1]Department of Biomedical Sciences, University of Padova, Padova 35121, Italy. [2]Department of Protein Science, KTH Royal Institute of Technology, Stockholm SE-10691, Sweden. [3]Department of Information Engineering, University of Padua, Padova 35121, Italy. [4]Institute of Biomembranes, Bioenergetics and Molecular Biotechnologies, National Research Council (CNR-IBIOM), Bari, Italy

Supplementary information

Dataset preparation

To exploit the manually curated dataset of STRPs of RepeatsDB, we employed the most recent update, dated 2023-05-03. Comprising 9834 entries, each entry represents a manually curated repeat region. This dataset is linked to a total of 9448 PDB chains, 5628 PDB IDs, and 2473 UniProt IDs (**Table S1**). Every entry within RepeatsDB is classified into four hierarchical levels following Kajava's classification: Class, Topology, Fold, and Clan, with each subsequent level serving as a subcategory of its predecessor. In our endeavor, we aggregate entries based on the top two levels, namely Class and Topology, to delineate STRP types (**Figure S1**). After selecting STRP types with a sufficient number of entries to evaluate the software's performance, we addressed redundancy by randomly choosing one repeat region for each unique UniProt ID. Subsequently, we clustered at 30% of identity the sequences of the associated PDB chains for the selected samples and randomly chose one sample from each cluster. **Table S2** illustrates the final count of samples for each selected type, resulting in a total of 1225 positive samples.

RepeatsDB entries	Count
Repeat region	9533
PDB chain	9140
PDB ID	5462
UniProt ID	2473

Table S1. Number of repeat regions, PDB chains, PDB IDs, and UniProt IDs associated with the entries in the RepeatsDB dataset.

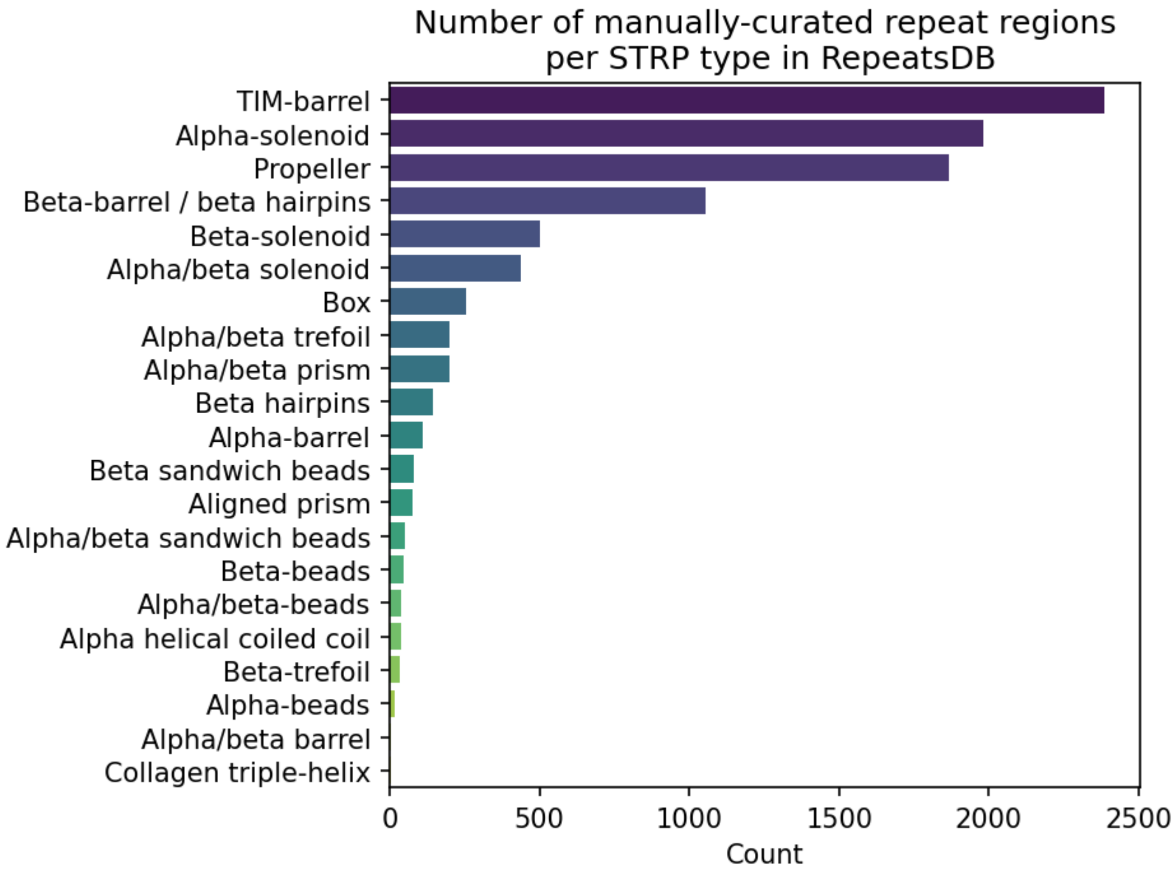

Figure S1. Number of entries per each type of STRPs in the RepeatsDB dataset.

Type	Count
Alpha-solenoid	401
Propeller	249
TIM-barrel	227
Beta-barrel	133
Beta-solenoid	120
Alpha/beta solenoid	95

Table S2. Number of unique entries per each type of STRP in the dataset used to evaluate the performance of the software.

Parameter optimization: “max_eval” and “min_height”

The “max_eval” parameter, which refers to the maximum allowed Foldseek E-value for filtering hits from the first round of alignment against the Tri-Unit-Library (TUL). It was optimized through a 5-fold cross-validation experiment. Five different values, ranging from 0.0001 to 1, were tested to determine the value that results in the highest F1-score, thus achieving the best balance between precision and recall.

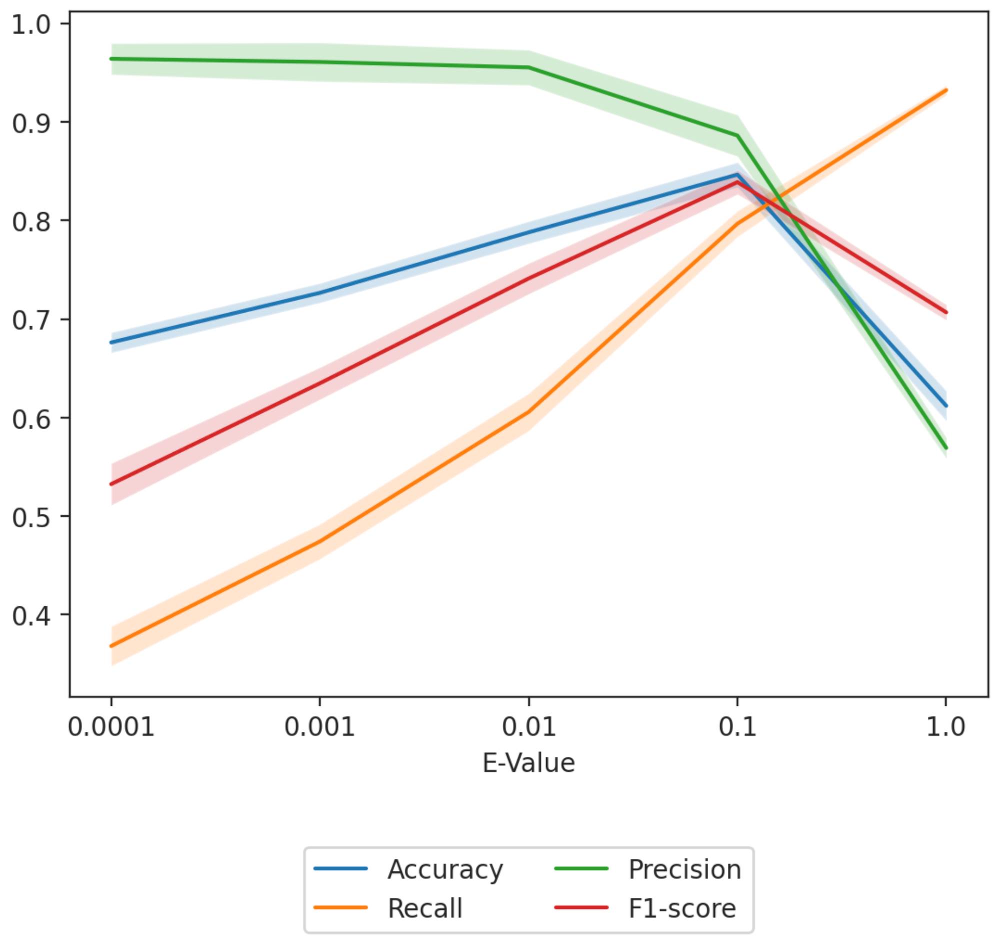

Figure S2. The impact of E-value maximum threshold on STRPsearch performance.

The “min_height” parameter, which defines the minimum peak height required for peak identification in the TM-score profile, was optimized using manual curations from RepeatsDB. For each manually curated repeat region in RepeatsDB, we aligned the repeat units within that region via multiple structure alignments and calculated the average structural similarity of the units in terms of TM-score. The mean and standard deviation of these average values were computed across different repeat types (class.topology). The lower end of one standard deviation below the mean was then used as the default “min_height” parameter for each repeat type. For example, if the identified representative unit from RUL is classified as Alpha-solenoid, a min_height parameter of 0.42 would be selected for the subsequent peak identification steps. This value is derived from the mean TM-score (0.56) minus one standard deviation (0.14) that was seen among the manually curated Alpha-solenoid repeat units in RepeatsDB.

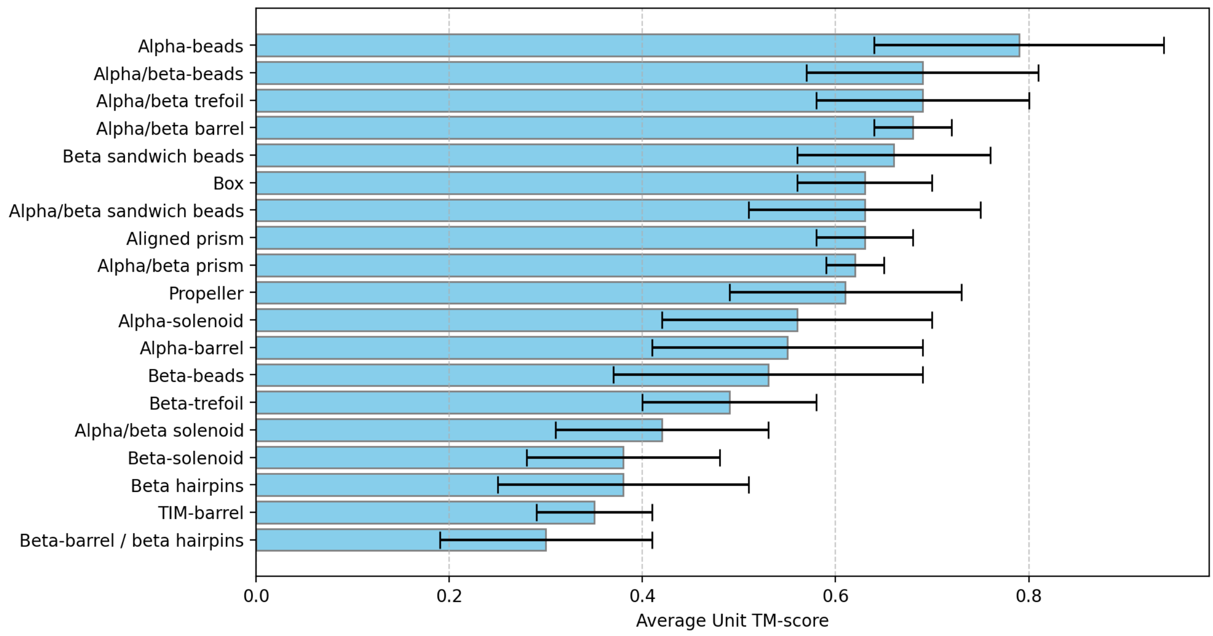

Figure S3. Average structural similarity of repeat units in different types of STRPs.

Cross-validation

Following a 5-fold cross-validation, the initial analysis focused on assessing the software's ability to distinguish STRPs from non-STRPs. To achieve this, the confusion matrix was computed for each fold. The raw counts of True Positives (TP), True Negatives (TN), False Positives (FP), and False Negatives (FN) for each fold are shown in **Table S3**. Additionally, performance metrics such as Accuracy, Precision, Recall, and F1-score were calculated to provide a more comprehensive evaluation of the software's performance (**Table S4**). In a separate analysis, we assessed the software's performance in detecting the range of repeat regions by evaluating its ability to identify residues within the curated range of each repeat region. The schematic representation in **Figure S2** shows the rationale behind this approach, and the **Table S5** presents the performance metrics for each fold. **Figure S3** shows the execution time per PDB chain by STRPsearch, RepeatsDB-Lite, and TAPO.

Fold	TP	FP	TN	FN
1	203	31	213	42
2	195	14	230	50
3	191	21	223	54
4	191	23	220	54
5	191	30	213	54

Table S3. Number of each component in the confusion matrix computed for each fold in the cross-validation test to evaluate the capability of the software in identifying STRPs from non-STRPs.

Fold	Accuracy	Precision	Recall	F1-Score
1	0.85	0.87	0.83	0.85
2	0.87	0.93	0.8	0.86
3	0.85	0.9	0.78	0.84
4	0.84	0.89	0.78	0.83
5	0.83	0.86	0.78	0.82

Table S4. The performance metrics, derived from the confusion matrix calculated for each fold during the cross-validation test, of the software in identifying STRPs from non-STRPs.

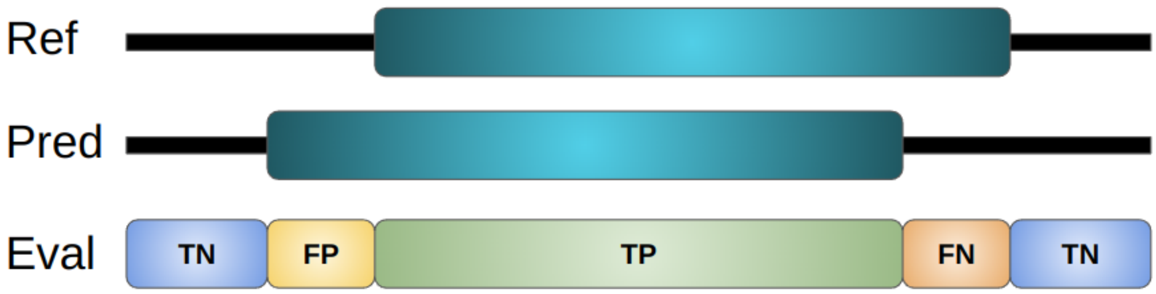

Figure S4. Schematic representation of the evaluative logic behind labeling the identified residues in the predictions compared to their associated reference.

Fold	Accuracy	Precision	Recall	F1-Score
1	0.88	0.91	0.91	0.9
2	0.89	0.92	0.93	0.91
3	0.89	0.93	0.91	0.91
4	0.87	0.9	0.91	0.89
5	0.86	0.9	0.91	0.89

Table S5. Performance metrics, calculated for each fold in the cross validation test, of the software in identifying STRP residues from non-STRP ones.

Benchmarking against with RepeatsDB-Lite and TAPO

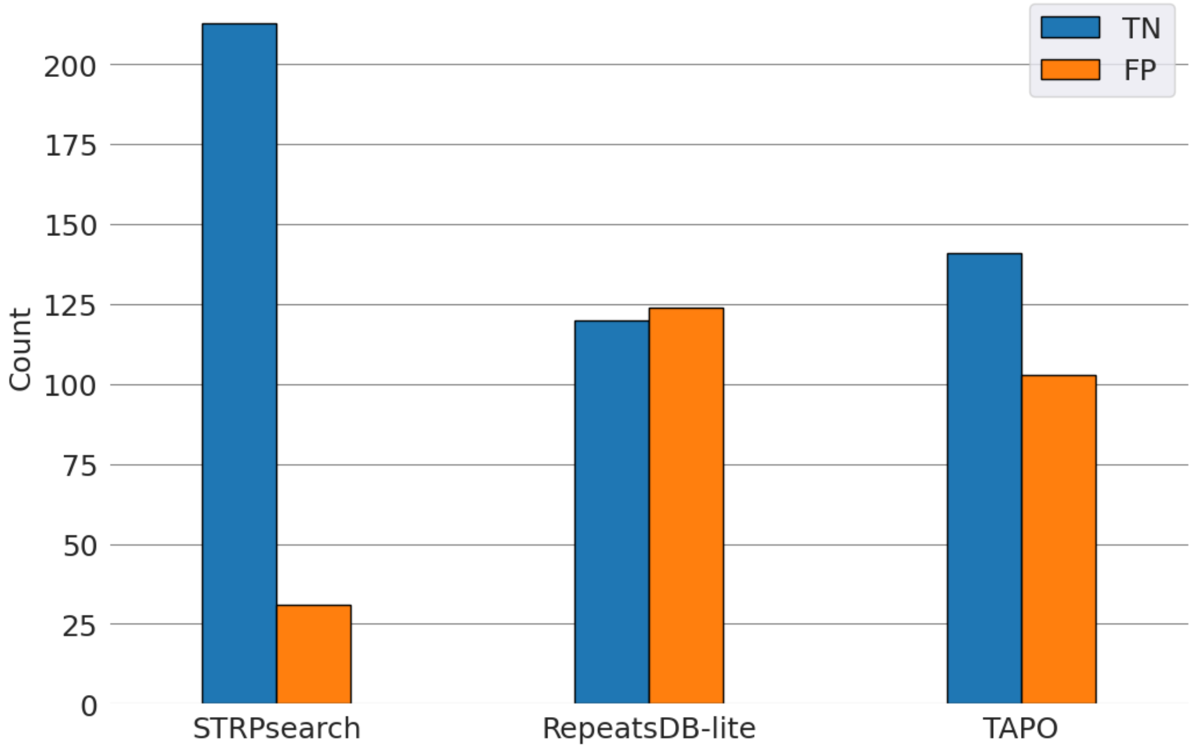

Figure S5. Benchmark comparison of the performance of different software when queried with negative structures. The blue bars represent true negatives (TN), indicating correctly identified non-STRP structures. The orange bars represent false positives (FP), indicating non-STRP structures incorrectly identified as STRPs.

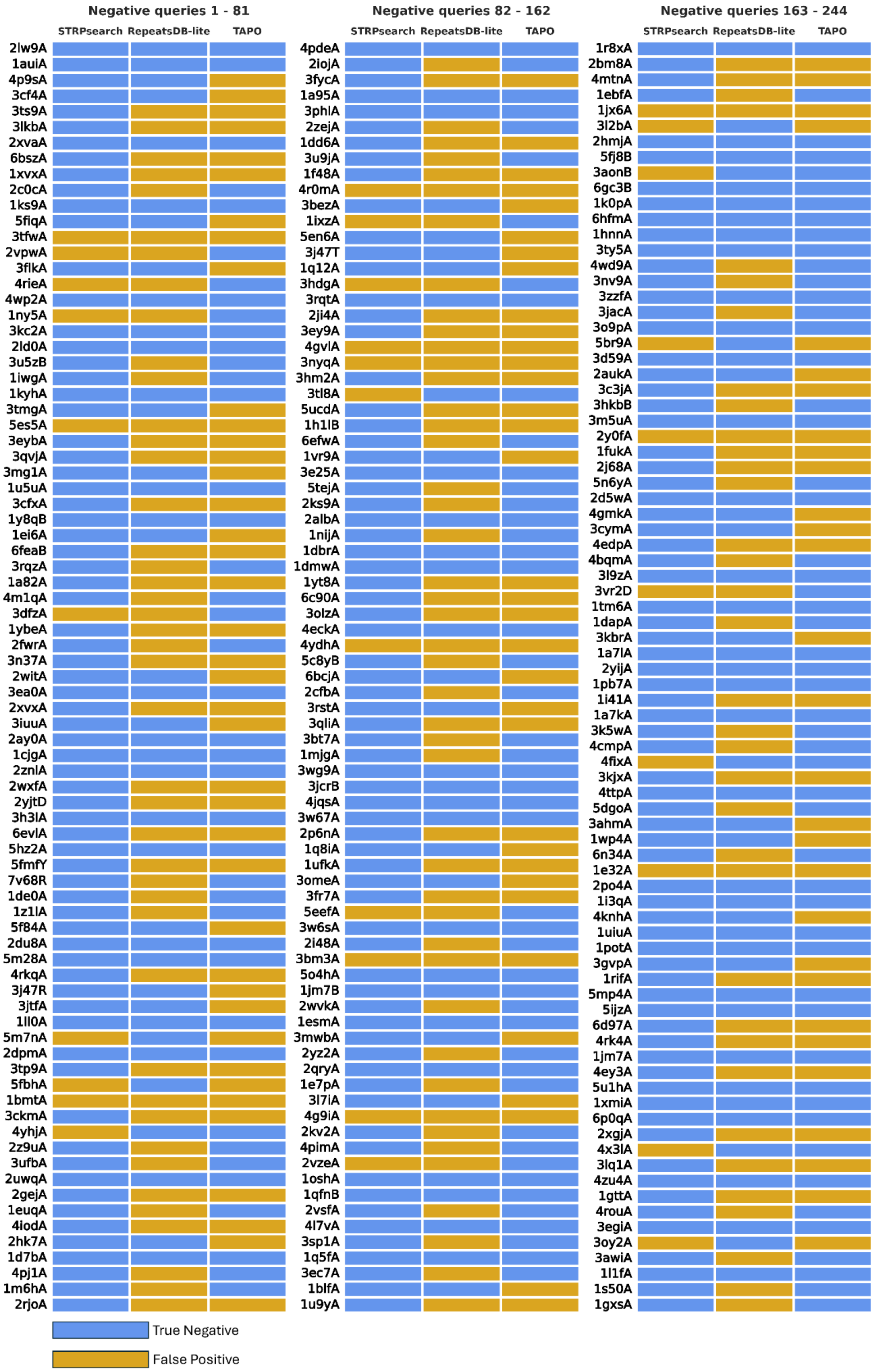

Figure S6. Detection of negative queries by STRPsearch, RepeatsDB-lite, and TAPO. This chart shows the detection results for negative queries (i.e., queries that do not contain STRPs) by STRPsearch, RepeatsDB-lite, and TAPO. Each row corresponds to a protein structure, and the columns show the detection outcomes by each software: blue indicates a correct negative detection (True Negative - TN), and orange indicates an incorrect positive detection (False Positive - FP).

Topology	STRPsearch	RepeatsDB-Lite	TAPO
Alpha-solenoid	0.86	0.95	0.97
Alpha/Beta solenoid	0.72	0.89	0.89
Beta-solenoid	0.42	0.75	0.92
Propeller	0.96	0.67	0.98
Beta-barrel	0.85	0.67	0.78
TIM-barrel	0.89	0.85	0.83

Table S6: Recall values comparison of STRPsearch, RepeatsDB-Lite and TAPO in detection of different STRP types.

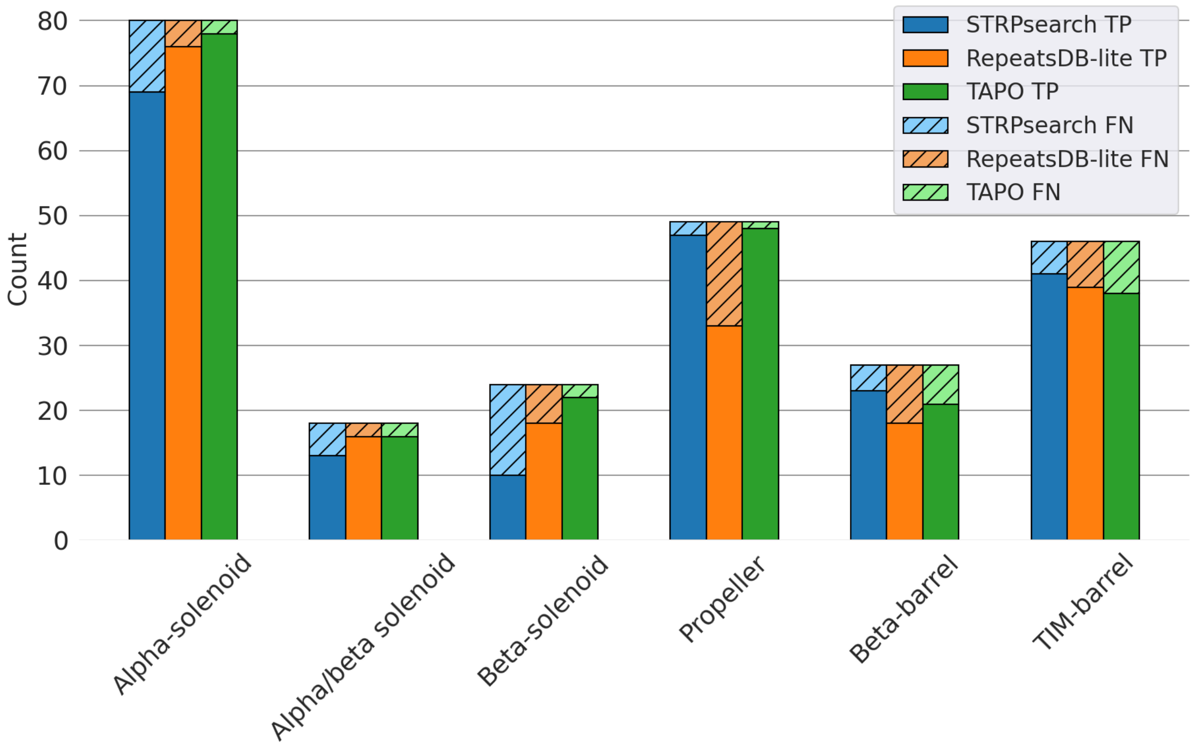

Figure S7. Benchmark comparison of the performance of STRPsearch, RepeatsDB-Lite and TAPO in detection of different STRP types when queried with positive structures. The solid colors (blue, orange, green) represent true positives (TP) for STRPsearch, RepeatsDB-lite, and TAPO, respectively. The striped sections represent false negatives (FN), indicating STRPs that were missed by the corresponding software.

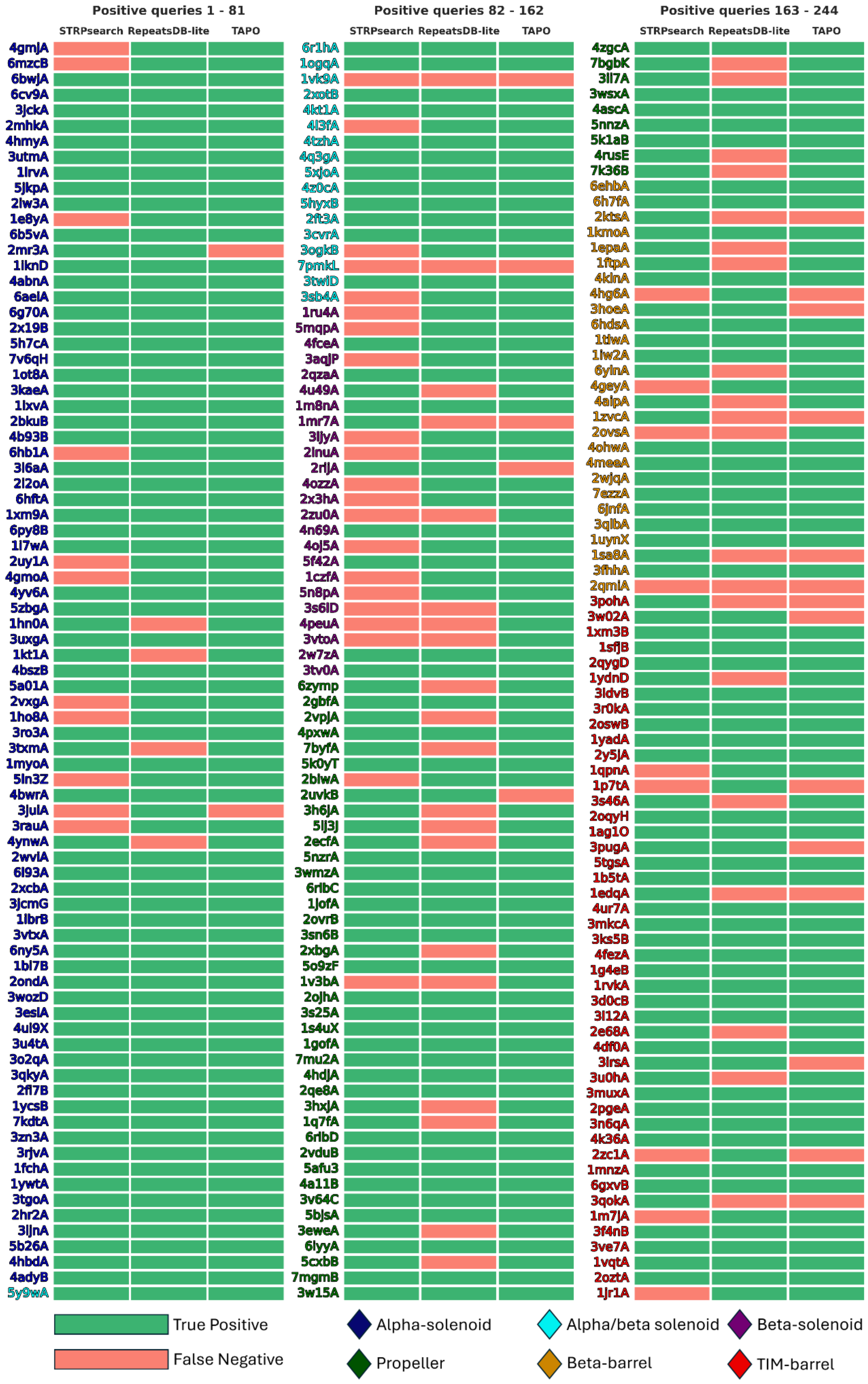
Positive queries 1 - 81
Positive queries 82 - 162
Positive queries 163 - 244
STRPsearch
RepeatsDB-lite
TAPO
4gmjA
6mzcB
6bwjA
6cv9A
3jckA
2mhkA
4hmyA
3utmA
1lrvA
5jkpA
2iw3A
1e8yA
6b5vA
2mr3A
1iknD
4abnA
6aelA
6g70A
2x19B
5h7cA
7v6qH
1ot8A
3kaeA
1ixvA
2bkuB
4b93B
6hb1A
3l6aA
2l2oA
6hftA
1xm9A
6py8B
1l7wA
2uy1A
4gmoA
4yv6A
5zbgA
1hn0A
3uxgA
1kt1A
4bszB
5a01A
2vxgA
1ho8A
3ro3A
3txmA
1myoA
5ln3Z
4bwrA
3julA
3rauA
4ynwA
2wvlA
6l93A
2xcbA
3jcmG
1lbrB
3vtxA
6ny5A
1bl7B
2ondA
3wozD
3eslA
4ul9X
3u4tA
3o2qA
3qkyA
2fl7B
1ycsB
7kdtA
3zn3A
3rjvA
1fchA
1ywtA
3tgoA
2hr2A
3ljnA
5b26A
4hbdA
4adyB
5y9wA
6r1hA
1ogqA
1vk9A
2xotB
4kt1A
4l3fA
4tzhA
4q3gA
5xjoA
4z0cA
5hyxB
2ft3A
3cvrA
3ogkB
7pmkL
3twiD
3sb4A
1ru4A
5mqpA
4fceA
3aqjP
2qzaA
4u49A
1m8nA
1mr7A
3ljyA
2inuA
2rljA
4ozzA
2x3hA
2zu0A
4n69A
4oj5A
5f42A
1czfA
5n8pA
3s6lD
4peuA
3vtoA
2w7zA
3tv0A
6zymp
2gbfA
2vpjA
4pxwA
7byfA
5k0yT
2blwA
2uvkB
3h6jA
5lj3J
2ecfA
5nzrA
3wmzA
6rlbC
1jofA
2ovrB
3sn6B
2xbgA
5o9zF
1v3bA
2ojhA
3s25A
1s4uX
1gofA
7mu2A
4hdjA
2qe8A
3hxjA
1q7fA
6rlbD
2vduB
5afu3
4a11B
3v64C
5bjsA
3eweA
6lyyA
5cxbB
7mgmB
3w15A
4zgcA
7bgbK
3ll7A
3wsxA
4ascA
5nnzA
5k1aB
4rusE
7k36B
6ehbA
6h7fA
2ktsA
1kmoA
1epaA
1ftpA
4kinA
4hg6A
3hoeA
6hdsA
1tlwA
1iw2A
6yinA
4geyA
4aipA
1zvcA
2ovsA
4ohwA
4meeA
2wjqA
7ezzA
6jnfA
3qlbA
1uynX
1sa8A
3fhhA
2qmlA
3pohA
3w02A
1xm3B
1sfjB
2qygD
1ydnD
3ldvB
3r0kA
2oswB
1yadA
2y5jA
1qpnA
1p7tA
3s46A
2oqyH
1ag1O
3pugA
5tgsA
1b5tA
1edqA
4ur7A
3mkcA
3ks5B
4fezA
1g4eB
1rvkA
3d0cB
3l12A
2e68A
4df0A
3lrsA
3u0hA
3muxA
2pgeA
3n6qA
4k36A
2zc1A
1mnzA
6gxvB
3qokA
1m7jA
3f4nB
3ve7A
1vqtA
2oztA
1jr1A
True Positive
False Negative
Alpha-solenoid
Alpha/beta solenoid
Beta-solenoid
Propeller
Beta-barrel
TIM-barrel

Figure S8. Detection of positive queries by STRPsearch, RepeatsDB-lite, and TAPO.
This chart displays the detection results for positive queries (queries that contain Structured Tandem Repeat Proteins - STRPs) by three different software: STRPsearch, RepeatsDB-lite, and TAPO. Each row represents a protein structure and the index is color-coded based on the STRP type it contains, and the columns represent the detection results by each software.

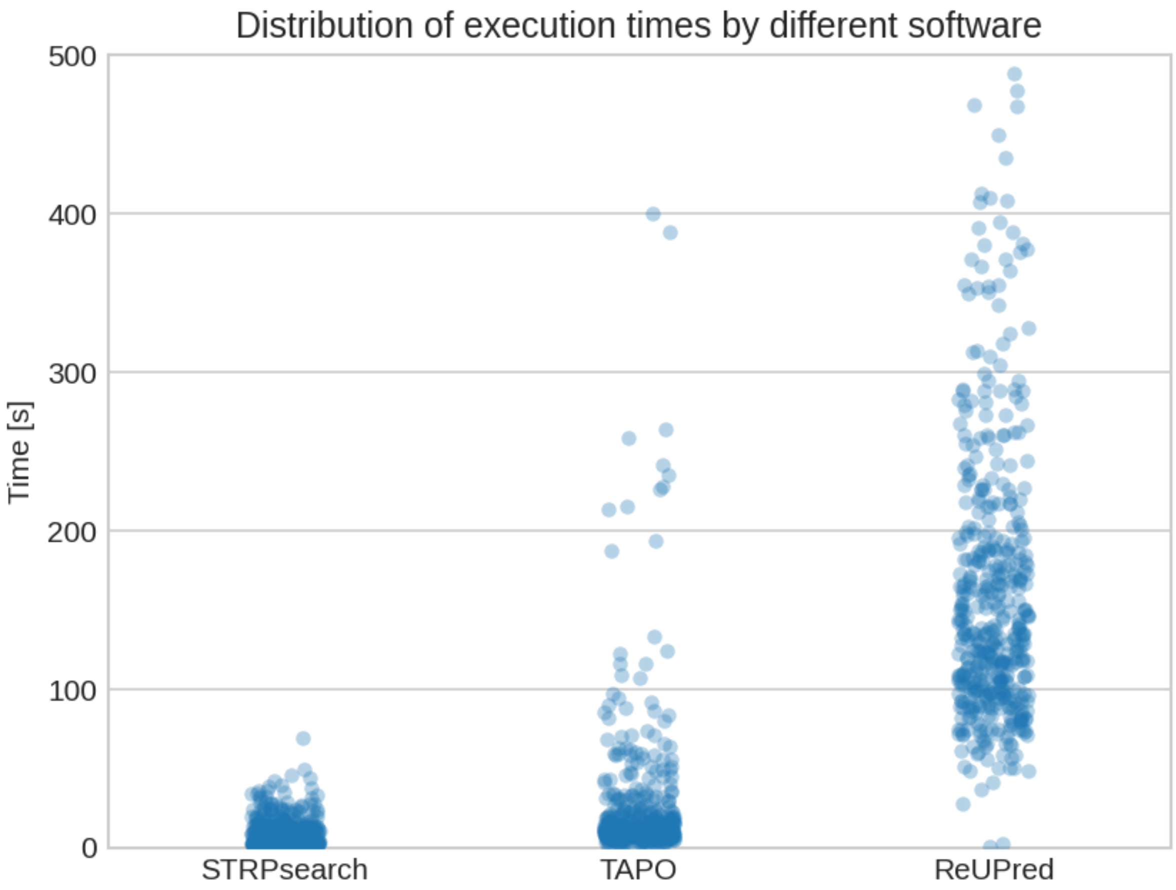

Figure S9. Distribution of execution times by different softwares on the benchmark dataset.
